# Changes in primary care management of atrial fibrillation patients following the shift from warfarin to non-vitamin K antagonist oral anticoagulants: a Norwegian population based study

**DOI:** 10.1186/s12875-022-01824-6

**Published:** 2022-08-25

**Authors:** Sigrun Halvorsen, Jørgen Anton Smith, Fabian Söderdahl, Marcus Thuresson, Oddvar Solli, Maria Ulvestad, Christian Jonasson

**Affiliations:** 1 Department of Cardiology, Oslo University Hospital Ullevål, Nydalen, Postboks 4956, 0424 Oslo, Norway; 2 University of Oslo, Oslo, Norway; 3 Rykkinn Legekontor (Rykkinn Medical Office), Rykkinn, Norway; 4 Statisticon AB, Uppsala, Sweden; 5 Health and Value Lead, Pfizer AS, Oslo, Norway; 6 Bristol Myers Squibb, Lysaker, Norway; 7 K. G. Jebsen Center for Genetic Epidemiology, Faculty of Medicine and Health Sciences, NTNU–Norwegian University of Science and Technology, Trondheim, Norway

**Keywords:** Non-vitamin K antagonist oral anticoagulants, Atrial fibrillation, Norway, Drug utilisation study, Primary care

## Abstract

**Background:**

To assess baseline characteristics, drug utilisation and healthcare use for oral anticoagulants (OACs) following the introduction of non-vitamin K antagonist oral anticoagulants among patients with atrial fibrillation in primary care in Norway.

**Methods:**

In this retrospective longitudinal cohort study, 92,936 patients with atrial fibrillation were identified from the Norwegian Primary Care Registry between 2010 and 2018. Linking to the Norwegian Prescription Database, we identified 64,112 patients (69.0%) treated with OACs and 28,824 (31%) who were untreated. Participants were followed until 15 May 2019, death, or loss to follow-up, whichever came first. For each OAC, predictors of initiation were assessed by modelling the probability of initiating the OAC using logistic regression, and predictors of the first switch after index date were assessed using multivariable Cox proportional hazards models. The numbers of primary care visits per quarter by index OAC were plotted and analysed with negative binomial regression analyses offset for the log of days at risk.

**Results:**

Patients treated with OACs were older, had more comorbidities, and higher CHA_2_DS_2_-VASc scores than untreated patients. However, the mean CHA_2_DS_2_-VASc in the non-OAC group was 1.58 for men and 3.13 for women, suggesting an indication for OAC therapy. The percentage of patients with atrial fibrillation initiating OACs increased from 59% in 2010 to 79% in 2018. Non-vitamin K antagonist oral anticoagulant use increased throughout the study period to 95% of new OAC-treated patients in 2018, and switches from warfarin to non-vitamin K antagonist oral anticoagulants were common. The persistence of OAC treatment was > 60% after four years, with greatest persistence for apixaban. Patients treated with non-vitamin K antagonist oral anticoagulants had fewer primary care visits compared with those treated with warfarin (incidence rate ratio: 0.73, 95% confidence interval 0.71 to 0.75).

**Conclusion:**

In this Norwegian primary care study, we found that the shift from warfarin to non-vitamin K antagonist oral anticoagulants was successful with 95% use in patients initiating OACs in 2018, and associated with fewer general practitioner visits. Persistence with OACs was high, particularly for apixaban. However, many patients eligible for treatment with OACs remained untreated.

**Supplementary Information:**

The online version contains supplementary material available at 10.1186/s12875-022-01824-6.

## Introduction

In the European Union, 8.8 million adults aged > 55 years were diagnosed with atrial fibrillation (AF) in 2010; this number is projected to double by 2060 due to the ageing population [1]. AF increases the risk for thromboembolic events, especially ischaemic strokes [2]. Warfarin and other vitamin K antagonists were traditionally established as effective treatments, reducing the risk of stroke by about two-thirds [3]. In recent years, four non-vitamin K antagonist oral anticoagulants (NOACs) (apixaban, dabigatran, edoxaban, and rivaroxaban) have been introduced as replacements for warfarin in patients with AF; they reduce routine laboratory monitoring, have fewer drug and food interactions, and have a more rapid onset and offset of action compared with warfarin [4–11]. The body of evidence from clinical trials and meta-analyses has shown similar or superior efficacy of NOACs in stroke prevention compared with warfarin and an association with a similar or reduced risk of bleeding [4, 6, 8–13]. Nationwide observational studies have also confirmed randomised controlled trial data on the safety and efficacy of NOACs in clinical practice [14–16].

Due to their advantages, NOACs have been quickly introduced in European guidelines for the treatment of AF [17, 18], and the rapid introduction has been well-handled by physicians and other relevant stakeholders. In Norway, NOACs have been reimbursed since 2013 and a shift was observed in market shares from complete warfarin coverage in 2010 to more than 90% of new NOAC users in 2015 [14, 19–21]. Recent review studies have indicated reduced healthcare resource use and improved clinical outcomes of NOACs compared with warfarin [22, 23]. However, most of the previous observational studies have identified AF cases from a secondary care hospital setting, and there is a knowledge gap on how shifting to NOACs affects patients in primary care. This study aimed to assess baseline characteristics, drug utilisation patterns, and primary healthcare use for oral anticoagulants (OACs) following the introduction of NOACs among patients with AF, using Norwegian population-based nationwide registries from 2010–2018.

## Methods

### Data sources

Two population-based registers with nationwide coverage were used: the Norwegian Primary Care Registry (KUHR) and the Norwegian Prescription Database (NorPD) [24]. The KUHR contains diagnoses codes (International Classification of Primary Care [ICPC]-2, International Classification of Diseases [ICD]-10) and claim codes from all primary care consultations since 2006, as well as patient demographics. The NorPD covers all prescriptions dispensed from pharmacies since 2004, using the Anatomical Therapeutic Chemical system. Data from the two registries were linked using the unique 11-digit national identification number. Data from KUHR and NorPD are considered high quality; however, some quality checking and data cleaning were done to check for reasonability and consistency of data.

### Cohort creation and study design

This was a retrospective longitudinal cohort study. The source population comprised all adult patients with AF registered in KUHR in the identification period, defined as 1 January 2010 to 31 December 2018. These data were linked to the NorPD to identify patients with and without OAC use (Cohort Creation Chart; Additional File 1). Participants were followed until 15 May 2019, death, or loss to follow-up, whichever came first. AF was defined as AF in the absence of rheumatic mitral stenosis, a mechanical or bioprosthetic heart valve, or mitral valve repair in accordance with the European Society of Cardiology 2020 AF guidelines [25].

Adult patients (age ≥ 18) with a diagnosis of AF or flutter (ICPC-2 code K78 or ICD-10 code I48x from KUHR) were identified and stratified based on OAC use during the study period. Patients treated with an OAC had to be diagnosed with AF or flutter during the 12-month period before to six months after the first OAC prescription date*,* which was considered their index date.

For non-OAC patients, the first diagnosis of AF/flutter during the study period was used as the index date, as by definition, non-OAC patients did not have a first OAC prescription date.

**Fig. 1:**
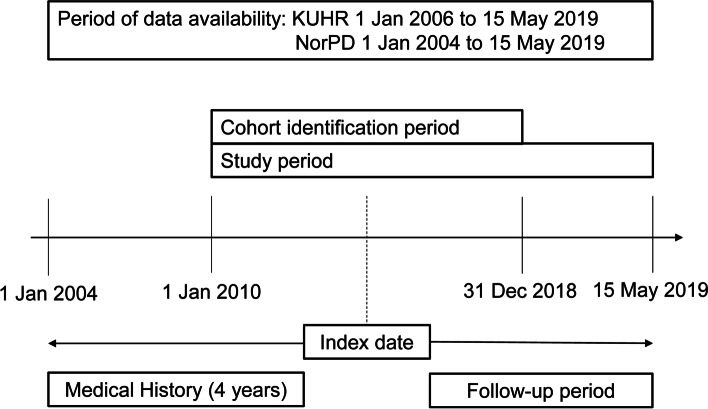
Study Design: Index Dates and Follow-up Periods. Abbreviations: KUHR = Norwegian Primary Care Registry; NorPD = Norwegian Prescription Database

Patients were excluded if they met any of the following criteria: presence of valvular disease, venous thromboembolism (VTE), or pregnancy during the 12-month period preceding the index date for OAC-treated patients and during the study period for non-OAC-treated patients. Also, patients with hip/knee replacement surgery within six weeks prior to index date for OAC-treated patients and during the study period for non-OAC-treated patients were excluded. Only incident OAC users were included; hence, OAC-treated patients were also excluded if they had an OAC dispensation during the 12 months preceding the OAC index date, or two or more different OACs dispensed on the index date. Figure 1 illustrates the study period, index dates, and follow-up periods for the cohort. For ICD codes for the conditions, see Additional File 2.

### Variables and definitions

#### Baseline patient characteristics

*Index year* was the year of the first OAC dispensation date for treated patients and year of first diagnosis for untreated patients. *Age* was measured at the index date, and *sex* was defined as male or female. *Medical history*, was assessed during the four years prior to the index date, including *prior stroke* and *prior major bleeding*. *Co-medications* were assessed from one year prior to index date to 120 days post index date. Based on the medical history and co-medications, the following scores were calculated: *Modified HAS-BLED score* (0–8) [26], the *CHA*_*2*_*DS*_*2*_*-VASc*(0–9 calculated separately for women and men) [27], and *the Comorbidity Index* (0–26). See Additional File 2 for a description of the scores and relevant codes.

#### OAC switch and discontinuation

Among treated patients, OAC drug exposure was assumed to be 100 days for each prescription. An allowable gap (grace period) between successive prescriptions of the same drug in calculating continuous exposure was set to be 100 days or less. *OAC switch* was defined as the first event of changing type of OAC, and *OAC discontinuation* was defined as time from the index OAC prescription to stopping treatment (independent of OAC switch). An alternative definition of *OAC discontinuation*—time from the index OAC prescription to the first switch or stopping treatment—was also explored in Kaplan–Meier curves.

#### Healthcare resource use during follow-up

The frequency of primary care consultations for the treated population was assessed during the entire period after the OAC index date and grouped into the following categories *ordinary primary care visits, laboratory work-up, electrocardiogram (ECG), and simple patient contact (such as a telephone consultation or equivalent)*, as well as the *total number of visits*. Consultations were defined using claims codes, see Additional File 2.

### Statistical analyses

Descriptive statistics for all baseline demographic and clinical characteristics were reported for OAC users and non-users, as well as by individual OACs. Continuous variables were summarised by their means and standard deviations, and categorical variables were summarised by the counts and proportions of patients in each category. Standardised mean difference (SMD) was used to assess balance in baseline characteristics between groups. No imputations for missing data were performed. Summary statistics were based on the non-missing (available) cases for each variable. *P*-values < 0.05 were considered statistically significant, and 95% confidence intervals (CI) were used. Confounders to be included in each model were selected a priori and included those suspected to be valuable predictors, based on previous research.

#### Predictors of OAC Initiation

For each OAC, predictors of initiation were assessed by modelling the probability of initiating the OAC using logistic regression. A fitted multivariable logistic regression model was used to model the probability of receiving NOAC therapy compared with warfarin, using warfarin as reference for the NOACs. Predictive performance of the final model was assessed by the c-statistic. Adjusted odds ratios were reported. Predictors were described from 2013 onwards for warfarin, rivaroxaban, and dabigatran and 2014 onwards for apixaban, as apixaban was approved for reimbursement six months later than rivaroxaban and dabigatran in Norway [14].

#### OAC dosing at initiation

Dose at OAC index date prescribed for AF was described by standard and reduced NOAC dose, by index year. A standard dose of dabigatran was considered 150 mg twice daily (bid), rivaroxaban 20 mg once daily, and apixaban 5 mg bid. Dabigatran < 150 mg bid, rivaroxaban < 20 mg once daily, and apixaban < 5 mg bid were considered reduced doses.

#### OAC switch

For each OAC, predictors of the first switch after index date were assessed using multivariable Cox proportional hazards models. Adjusted hazard ratios (HR) were reported. Cumulative incidence curves for OAC switches adjusted for competing risk were also presented.

#### OAC discontinuation

Predictors of OAC discontinuation were assessed using multivariable Cox proportional hazards models. Adjusted HRs were reported. Kaplan–Meier curves for time to discontinuation for each OAC, and overall discontinuation for all NOACs, were also presented.

#### Healthcare resource use during follow-up

The numbers of visits per quarter by index OAC were plotted and analysed with negative binomial regression analyses offset for the log of days at risk. Adjusted incidence rate ratios (IRR) were reported.

OAC switch, discontinuation, and healthcare resource use were described for patients with index years 2015 onwards, as dabigatran, rivaroxaban, and apixaban were all approved for reimbursement and in use by Norwegian healthcare providers. Predictors for switch and discontinuation were analysed for the year 2017, as these drugs were all well established and in frequent use by then.

## Results

### Baseline characteristics

We identified 92,936 patients with AF, with a mean follow-up of 4.3 years (276,673 years of follow-up in total). Of these, 64,112 (69.0%) were treated with an OAC and 28,824 (31%) were not treated with OACs (Table 1). Patients in the OAC group were older and had higher mean CHA_2_DS_2_-VASc, Modified HAS-BLED, and Comorbidity Index scores compared with the non-OAC group (Table 1), although standardized mean differences were small (Table 1). The mean CHA_2_DS_2_-VASc score in the non-OAC group was 1.58 for men and 3.13 for women (Table 1), suggesting an indication for anticoagulation.

**Table 1:** Baseline Characteristics of Non-OAC and OAC Cohorts

Variable	Overall	Non-OAC	OAC	SMD
n	92,936	28,824	64,112	
Female, n (%)	40,960 (44.1)	13,317 (46.2)	27,643 (43.1)	0.062
Mean Age (SD)	71.31 (13.46)	67.66 (16.93)	72.95 (11.19)	0.368
**Concomitant medication (1-year preceding index to 120 days post index), n (%)**				
NSAID	24,623 (26.5)	8374 (29.1)	16,249 (25.3)	0.083
Anti-platelet treatment, including low-dose aspirin	47,108 (50.7)	14,218 (49.3)	32,890 (51.3)	0.039
Per-oral antidiabetic drugs	8482 (9.1)	1825 (6.3)	6657 (10.4)	0.147
Acid secretory drugs	25,861 (27.8)	7972 (27.7)	17,889 (27.9)	0.005
Heparin	5475 (5.9)	1625 (5.6)	3850 (6.0)	0.016
Anti-arrhythmic drugs class iii	6355 (6.8)	1268 (4.4)	5087 (7.9)	0.147
Anti-hypertensives	2049 (2.2)	422 (1.5)	1627 (2.5)	0.077
Diuretics	28,519 (30.7)	6829 (23.7)	21,690 (33.8)	0.225
Beta-blockers	67,830 (73.0)	16,848 (58.5)	50,982 (79.5)	0.468
Calcium antagonists	25,991 (28.0)	6507 (22.6)	19,484 (30.4)	0.178
Renin-angiotensin system drugs	46,452 (50.0)	10,330 (35.8)	36,122 (56.3)	0.420
Lipid-modifying drugs	39,870 (42.9)	9505 (33.0)	30,365 (47.4)	0.297
Insulin	2968 (3.2)	672 (2.3)	2296 (3.6)	0.074
**Medical history (4 years preceding index), n (%)**				
Alcoholism	1330 (1.4)	630 (2.2)	700 (1.1)	0.086
Chronic kidney disease	4088 (4.4)	1344 (4.7)	2744 (4.3)	0.019
Congestive heart failure	7703 (8.3)	2625 (9.1)	5078 (7.9)	0.043
Hypertension	46,280 (49.8)	11,825 (41.0)	34,455 (53.7)	0.257
Liver disease	911 (1.0)	350 (1.2)	561 (0.9)	0.033
Stroke	6588 (7.1)	1884 (6.5)	4704 (7.3)	0.032
Transient ischaemic attack	3863 (4.2)	1081 (3.8)	2782 (4.3)	0.030
Myocardial infarction	2797 (3.0)	910 (3.2)	1887 (2.9)	0.012
Angina Pectoris	8498 (9.1)	2617 (9.1)	5881 (9.2)	0.003
Peripheral artery disease	3077 (3.3)	854 (3.0)	2223 (3.5)	0.029
Pulmonary embolism	554 (0.6)	177 (0.6)	377 (0.6)	0.003
Prior major bleeding (critical organ)	6370 (6.9)	2325 (8.1)	4045 (6.3)	0.068
Type 2 diabetes	12,501 (13.5)	2856 (9.9)	9645 (15.0)	0.156
Dementia	2207 (2.4)	1167 (4.0)	1040 (1.6)	0.147
COPD	7989 (8.6)	2362 (8.2)	5627 (8.8)	0.021
Connective tissue disease	7234 (7.8)	2374 (8.2)	4860 (7.6)	0.024
Leukaemia	273 (0.3)	86 (0.3)	187 (0.3)	0.001
Lymphoma	720 (0.8)	264 (0.9)	456 (0.7)	0.023
Solid tumour	13,090 (14.1)	4126 (14.3)	8964 (14.0)	0.010
**Risk scores, mean (SD)**				
CHA2dsVASc men	1.97 (1.44)	1.58 (1.51)	2.14 (1.37)	0.394
CHA2dsVASc women	3.44 (1.41)	3.13 (1.58)	3.59 (1.29)	0.319
Modified HAS-BLED Score	2.04 (1.06)	1.81 (1.14)	2.14 (1.01)	0.301
Co-Morbidity Index	4.45 (2.04)	4.10 (2.36)	4.61 (1.85)	0.241

*Abbreviations*: *COPD* Chronic obstructive pulmonary disease, *NSAID* Non-steroidal anti-inflammatory drug, *OAC* Oral anticoagulant, *SD* Standard deviation, *SMD* Standardised mean difference

**Fig. 2:**
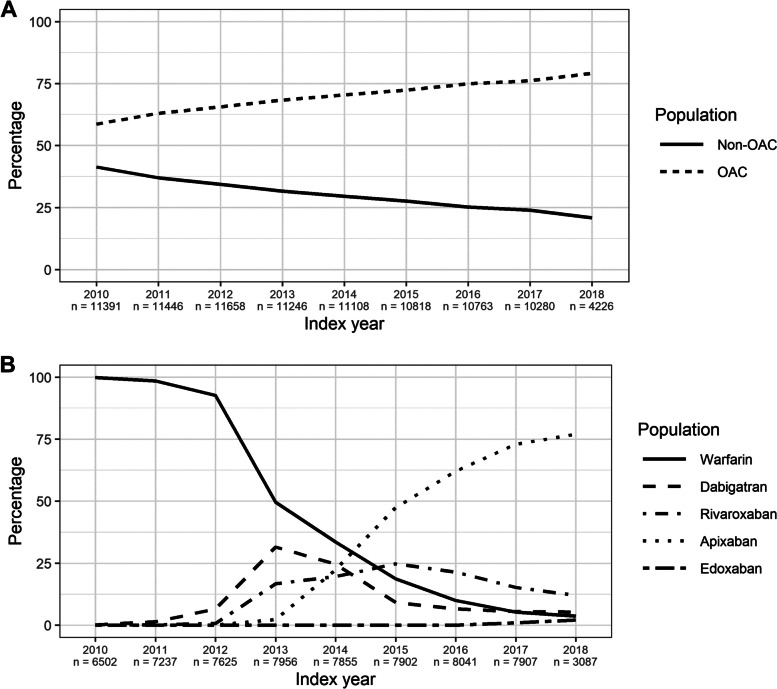
Percentage **a**) Non-OAC and OAC Patients by Index Year and **b**) OAC Patients by OAC Type and Index Year. Abbreviation: OAC = oral anticoagulant

The percentage of AF patients initiating OAC increased over time, from 59% in 2010 to 79% in 2018 (Fig. 2). In the earlier years of the study period, most OAC-treated patients received warfarin. However, from 2013 onwards, most OAC-naïve patients were started on a NOAC, and the proportion increased throughout the study period to 95% in 2018 (Fig. 2). Particularly, the number of patients starting on apixaban increased steadily from 2013 to 2018.

We identified 30,068 patients with AF (46.9%) with a first prescription for warfarin in the study period, 18,815 patients (29.3%) with a first prescription for apixaban, 8,191 patients (12.8%) with a first prescription for rivaroxaban, and 6,896 patients (10.8%) with a first prescription for dabigatran. Switching from warfarin to the NOACs was common (see OAC Switch results section), so the real number treated with NOACs was higher. Edoxaban was introduced later than the other drugs, with its first index date in 2016, and was used by 142 patients (0.2%) (Table 2). Because of the low uptake, edoxaban was excluded from the regression models. There were no large differences in baseline characteristics between patients initiating the different OACs. The proportion of women ranged from 40.2% on dabigatran to 46.5% on edoxaban, and the mean age ranged from 70.76 on dabigatran to 73.40 on apixaban (Table 2). There were no large differences in the mean CHA_2_DS_2_-VASc, Modified HAS-BLED, and Comorbidity Index scores between the OACs (SMD 0.069 to 0.139) (Table 2).

**Table 2:** Baseline Characteristics of the OAC Cohort, by Separate OACs

Variable	Overall	Warfarin	Dabigatran	Rivaroxaban	Apixaban	Edoxaban	SMD
n	64,112	30,068	6896	8191	18,815	142	
Female n (%)	27,643 (43.1)	12,540 (41.7)	2772 (40.2)	3628 (44.3)	8637 (45.9)	66 (46.5)	0.068
Mean Age (SD)	72.91 (11.18)	73.14 (11.21)	70.76 (11.21)	72.74 (11.01)	73.40 (11.09)	73.06 (9.59)	0.104
**Concomitant medication (1-year preceding index to 120 days post index), n (%)**							
NSAID (%)	16,568 (25.8)	7869 (26.2)	1920 (27.8)	2122 (25.9)	4623 (24.6)	34 (23.9)	0.043
Anti-platelet treatment, including low-dose aspirin (%)	32,940 (51.4)	17,361 (57.7)	3128 (45.4)	3808 (46.5)	8586 (45.6)	57 (40.1)	0.147
Per-oral antidiabetic drugs (%)	6667 (10.4)	3348 (11.1)	625 (9.1)	800 (9.8)	1881 (10.0)	13 (9.2)	0.033
Acid secretory drugs (%)	17,964 (28.0)	8166 (27.2)	1780 (25.8)	2230 (27.2)	5742 (30.5)	46 (32.4)	0.073
Heparin (%)	3884 (6.1)	2640 (8.8)	233 (3.4)	339 (4.1)	669 (3.6)	3 (2.1)	0.129
Anti-arrhythmic drugs class iii (%)	5090 (7.9)	2177 (7.2)	459 (6.7)	538 (6.6)	1904 (10.1)	12 (8.5)	0.065
Anti-hypertensives (%)	1634 (2.5)	881 (2.9)	143 (2.1)	162 (2.0)	444 (2.4)	4 (2.8)	0.034
Diuretics (%)	21,735 (33.9)	11,960 (39.8)	1832 (26.6)	2267 (27.7)	5638 (30.0)	38 (26.8)	0.127
Beta-blockers (%)	51,007 (79.6)	24,217 (80.5)	5461 (79.2)	6136 (74.9)	15,097 (80.2)	96 (67.6)	0.145
Calcium antagonists (%)	19,527 (30.5)	9761 (32.5)	1830 (26.5)	2386 (29.1)	5508 (29.3)	42 (29.6)	0.054
Renin-angiotensin system drugs (%)	36,163 (56.4)	17,344 (57.7)	3685 (53.4)	4487 (54.8)	10,574 (56.2)	73 (51.4)	0.062
Lipid-modifying drugs (%)	30,423 (47.5)	14,895 (49.5)	2972 (43.1)	3680 (44.9)	8809 (46.8)	67 (47.2)	0.061
Insulin (%)	2299 (3.6)	1284 (4.3)	153 (2.2)	234 (2.9)	627 (3.3)	1 (0.7)	0.109
**Medical history (4 years preceding index), n (%)**							
Alcoholism (%)	705 (1.1)	300 (1.0)	85 (1.2)	91 (1.1)	225 (1.2)	4 (2.8)	0.056
Chronic kidney disease (%)	2750 (4.3)	1513 (5.0)	147 (2.1)	249 (3.0)	837 (4.4)	4 (2.8)	0.081
Congestive heart failure (%)	5089 (7.9)	2874 (9.6)	401 (5.8)	526 (6.4)	1278 (6.8)	10 (7.0)	0.061
Hypertension (%)	34,529 (53.9)	16,090 (53.5)	3640 (52.8)	4507 (55.0)	10,221 (54.3)	71 (50.0)	0.046
Liver disease (%)	567 (0.9)	246 (0.8)	56 (0.8)	76 (0.9)	187 (1.0)	2 (1.4)	0.027
Stroke (%)	4720 (7.4)	2458 (8.2)	428 (6.2)	540 (6.6)	1283 (6.8)	11 (7.7)	0.039
Transient ischaemic attack (%)	2789 (4.4)	1414 (4.7)	242 (3.5)	342 (4.2)	788 (4.2)	3 (2.1)	0.065
Myocardial infarction (%)	1897 (3.0)	1055 (3.5)	158 (2.3)	185 (2.3)	494 (2.6)	5 (3.5)	0.045
Angina Pectoris (%)	5906 (9.2)	3251 (10.8)	472 (6.8)	624 (7.6)	1551 (8.2)	8 (5.6)	0.087
Peripheral artery disease (%)	2232 (3.5)	1166 (3.9)	194 (2.8)	258 (3.1)	607 (3.2)	7 (4.9)	0.052
Pulmonary embolism (%)	378 (0.6)	253 (0.8)	25 (0.4)	38 (0.5)	62 (0.3)	0 (0.0)	0.061
Prior major bleeding (critical organ) (%)	4078 (6.4)	1882 (6.3)	393 (5.7)	502 (6.1)	1288 (6.8)	13 (9.2)	0.059
Type 2 diabetes (%)	9660 (15.1)	4926 (16.4)	904 (13.1)	1152 (14.1)	2664 (14.2)	14 (9.9)	0.084
Dementia (%)	1042 (1.6)	449 (1.5)	77 (1.1)	153 (1.9)	359 (1.9)	4 (2.8)	0.056
COPD (%)	5648 (8.8)	2692 (9.0)	505 (7.3)	664 (8.1)	1776 (9.4)	11 (7.7)	0.039
Connective tissue disease (%)	4888 (7.6)	2245 (7.5)	511 (7.4)	631 (7.7)	1488 (7.9)	13 (9.2)	0.029
Leukaemia (%)	187 (0.3)	80 (0.3)	16 (0.2)	23 (0.3)	68 (0.4)	0 (0.0)	0.038
Lymphoma (%)	458 (0.7)	234 (0.8)	37 (0.5)	57 (0.7)	129 (0.7)	1 (0.7)	0.012
Solid tumour (%)	8999 (14.0)	3977 (13.2)	892 (12.9)	1169 (14.3)	2934 (15.6)	27 (19.0)	0.080
**Risk scores, mean (SD)**							
CHA2dsVASc men	2.14 (1.37)	2.22 (1.41)	1.90 (1.32)	2.12 (1.34)	2.12 (1.33)	2.11 (1.20)	0.099
CHA2dsVASc women	3.59 (1.29)	3.68 (1.33)	3.40 (1.28)	3.47 (1.22)	3.56 (1.26)	3.32 (1.25)	0.139
Modified HAS-BLED Score	2.14 (1.01)	2.19 (1.00)	2.03 (1.04)	2.11 (0.99)	2.12 (1.01)	2.09 (1.00)	0.069
Co-Morbidity Index	4.61 (1.86)	4.66 (1.87)	4.29 (1.79)	4.55 (1.81)	4.69 (1.87)	4.79 (1.86)	0.124

*Abbreviations*: *COPD* Chronic obstructive pulmonary disease, *NSAID* Non-steroidal anti-inflammatory drug, *OAC* Oral anticoagulant, *SD* Standard deviation, *SMD* Standardised mean difference

### Predictors for OAC initiation

The multivariable logistic regression model (including all predictors mentioned below) showed that, for every 10-year increase in age, there was a 12% decreased probability of dabigatran initiation compared with warfarin (odds ratio [OR]:0.88; 95% CI: 0.84–0.92). No statistically significant associations were observed between age and initiation of the other OAC types. Females were 52% more likely to initiate apixaban, 38% more likely to initiate rivaroxaban, and 36% more likely to initiate dabigatran compared with warfarin (OR:1.52; 95% CI: 1.41–1.64, OR: 1.38 95% CI: 1.29–1.49, OR: 1.36, 95% CI: 1.25–1.48, respectively). There was no statistically significant association between prior stroke or prior major bleeding and type of OAC initiation. A one-unit change in HAS-BLED score was associated with a 7% increased likelihood of initiating dabigatran compared with warfarin (OR:1.07; 95% CI: 1.02–1.12). No statistically significant associations were observed between HAS-BLED score and initiation of the other OAC types. A one-unit increase in CHA_2_DS_2_-VASc score was associated with decreases in the likelihood of initiation of rivaroxaban, dabigatran, and apixaban compared with warfarin (OR: 0.87; 95% CI: 0.83–0.90, OR: 0.83; 95% CI: 0.79–0.87, and OR: 0.86; 95% CI: 0.83–0.90, respectively).

### OAC dosing at initiation

More patients were treated with the standard dose compared with the reduced doses as the study years increased. Dabigatran 150 mg bid increased from 29.8% of patients treated in 2011 to 63.8% in 2018, rivaroxaban 20 mg once daily increased from 72.4% in 2012 to 81.4% in 2018, and apixaban 5 mg bid increased from 70.1% in 2013 to 81.1% in 2018 (Table 3).

**Table 3:** Index OAC Dose

Year	Warfarin	Dabigatran			Rivaroxaban				Apixaban	
**Dose**	**2.5**	**75**	**110**	**150**^**a**^	**2.5**	**10**	**15**	**20**^**a**^	**2.5**	**5**^**a**^
2010	6491 (100.00)	4 (44.44)	5 (55.56)	-	-	2 (100.00)	-	-	-	-
2011	7125 (100.00)	10 (9.62)	63 (60.58)	31 (29.81)	-	8 (100.00)	-	-	-	-
2012	7066 (100.00)	32 (6.40)	202 (40.40)	266 (53.20)	-	14 (24.14)	2 (3.45)	42 (72.41)	1 (100.00)	-
2013	3943 (100.00)	25 (1.00)	907 (36.25)	1570 (62.75)	-	46 (3.47)	353 (26.60)	928 (69.93)	55 (29.89)	129 (70.11)
2014	2633 (100.00)	11 (0.57)	565 (29.26)	1355 (70.17)	-	21 (1.37)	396 (25.75)	1121 (72.89)	453 (25.84)	1300 (74.16)
2015	1479 (100.00)	9 (1.24)	225 (31.08)	490 (67.68)	6 (0.31)	20 (1.02)	423 (21.63)	1507 (77.04)	868 (23.19)	2875 (76.81)
2016	801 (100.00)	5 (0.95)	171 (32.57)	349 (66.48)	4 (0.23)	10 (0.58)	284 (16.49)	1424 (82.69)	1101 (22.07)	3888 (77.93)
2017	416 (100.00)	1 (0.23)	154 (35.16)	283 (64.61)	1 (0.08)	13 (1.07)	219 (18.10)	977 (80.74)	1079 (18.70)	4690 (81.30)
2018	114 (100.00)	1 (0.61)	58 (35.58)	104 (63.80)	2 (0.54)	2 (0.54)	65 (17.57)	301 (81.35)	450 (18.94)	1926 (81.06)

Note: dose in milligrams, ^a^standard dose

### OAC switch

**Fig. 3:**
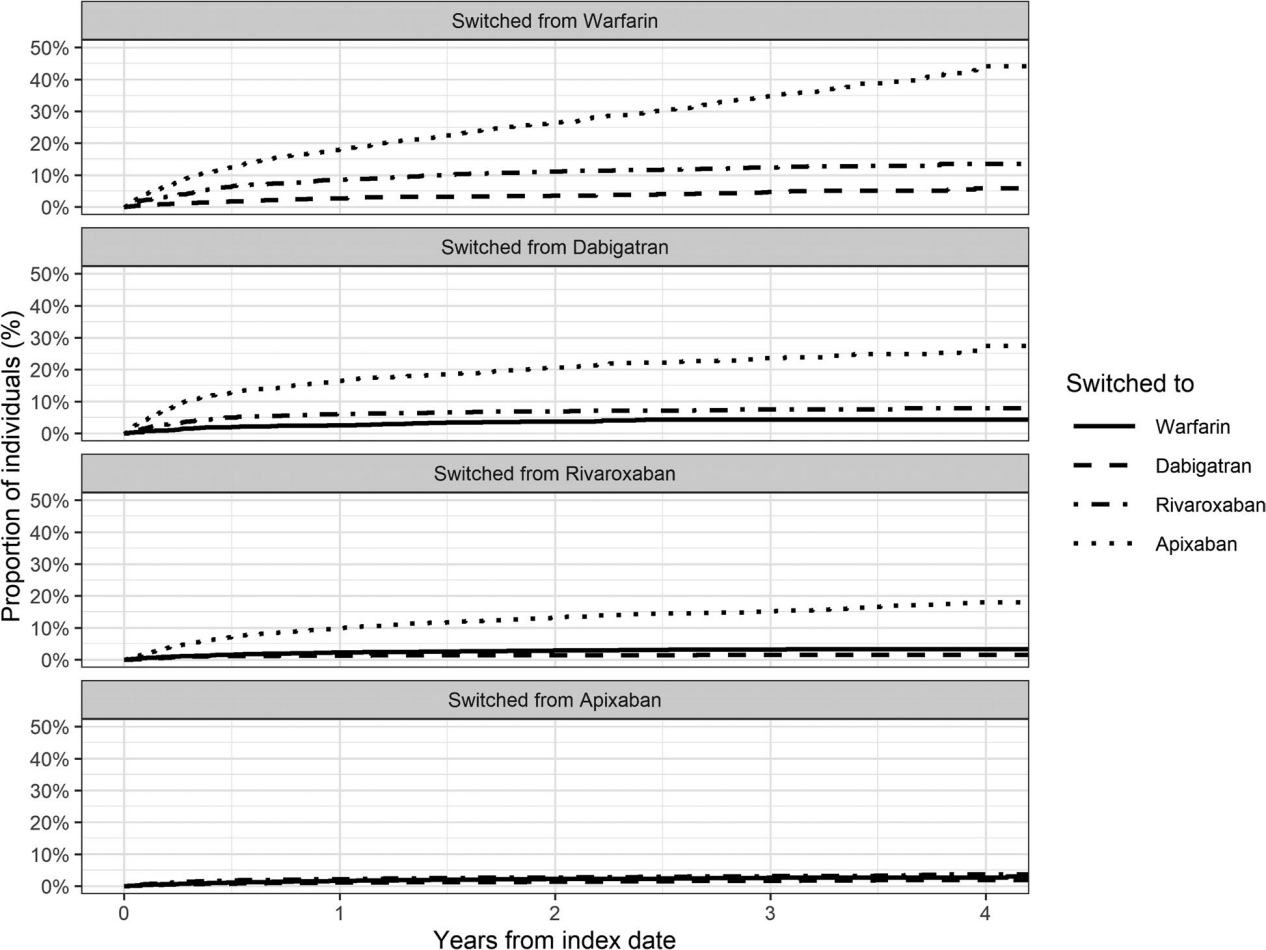
Cumulative Incidence for OAC Switches. Refer to Additional File 3 to see the ‘Switched from Apixaban’ graph with a y-axis scale up to 10%

There were frequent switches between OACs during the study period (Fig. 3), with most of those on warfarin switching to a NOAC between 2015 and 2018, most commonly apixaban. There were also frequent switches from dabigatran and rivaroxaban to apixaban during the study period (Fig. 3).

The multivariable Cox regression models (including all predictors mentioned below) concerning predictive factors for OAC switch in 2017 showed that a one-step increase in HAS-BLED score was associated with a higher risk of switching from rivaroxaban to another OAC (HR: 1.33; 95% CI: 1.06–1.66). There were no statistically significant associations between switch and age, sex, prior stroke, prior major bleeding, or CHA_2_DS_2_VASc score (numbers not shown).

### OAC discontinuation

**Fig. 4:**
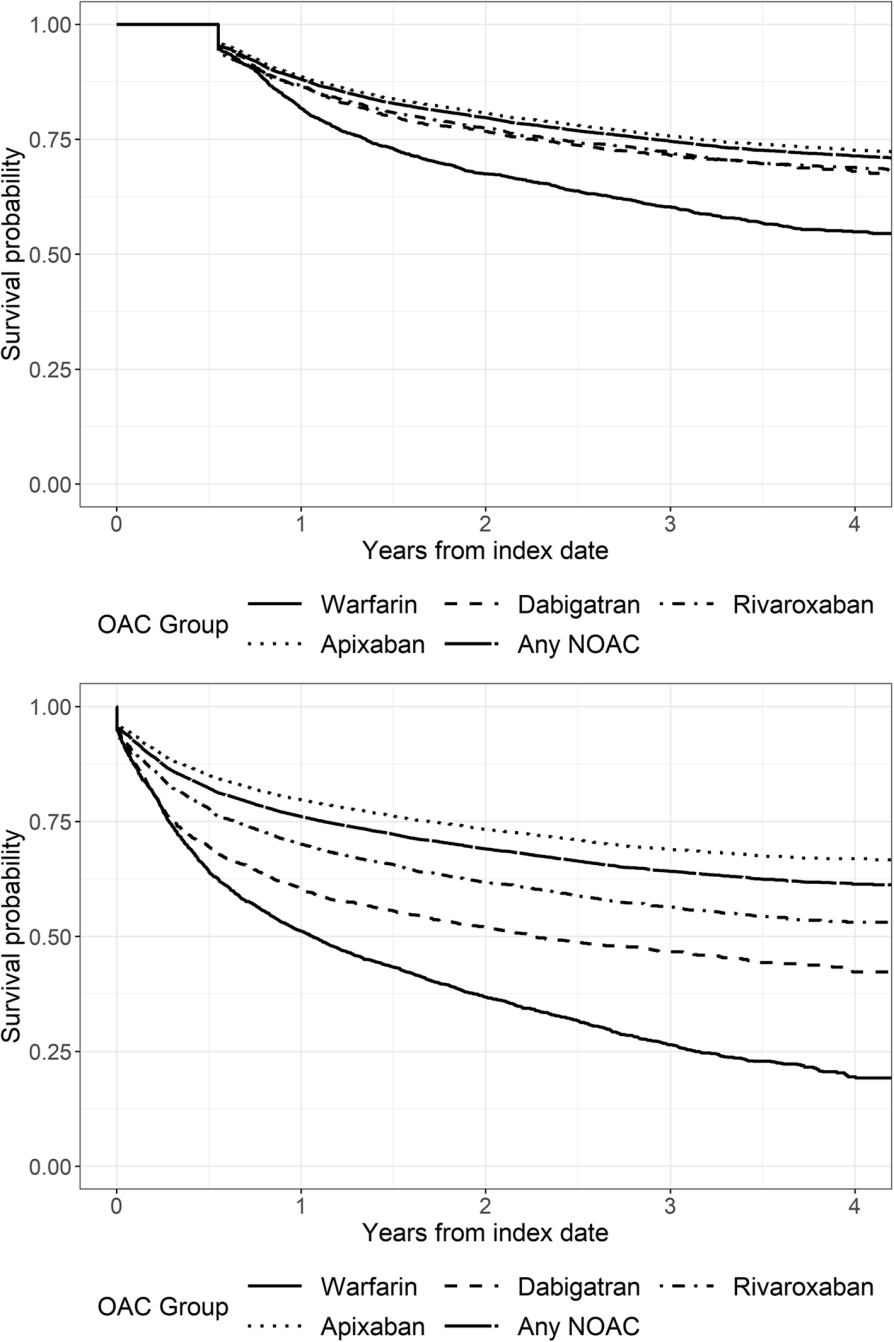
Kaplan–Meier Plot for Time to Discontinuation. Note: Time to discontinuation is defined as time from the index OAC prescription to stopping treatment (independent of OAC switch) (top graph) and time from the index OAC prescription to the first switch or stopping treatment (bottom graph). The minimum time to discontinuation independent of OAC switch is 200 days (100-day prescription and 100-day grace period). Abbreviations: OAC = oral anticoagulant; NOAC = non-vitamin K antagonist oral anticoagulant

With discontinuation defined as time from the index OAC prescription to stopping treatment (independent of OAC switch), the overall persistence for the OACs was more than 60% continuing OAC treatment for four years. With discontinuation defined as time from the index OAC prescription to the first switch or stopping treatment, the persistence was more than 40% for the NOACs and lower for warfarin. With either definition for discontinuation, those on apixaban were least likely to discontinue treatment; warfarin patients were most likely to discontinue treatment (Fig. 4).

The multivariable Cox regression models (including all predictors mentioned below) concerning predictive factors for OAC discontinuation (defined as first switch or stopping treatment) in 2017 showed higher age (10-year increase) was associated with a lower risk of discontinuation for rivaroxaban (HR: 0.82; 95% CI: 0.73–0.92) and apixaban (HR: 0.77; 95% CI: 0.72–0.82). Women were less likely to discontinue apixaban (HR: 0.80; 95% CI: 0.70–0.92). Prior major bleeds and prior stroke were not significantly associated with discontinuation. A one-point increase in HAS-BLED score was associated with a reduced risk of discontinuing apixaban (HR: 0.77; 95% CI: 0.71–0.84). A one-point increase in CHA_2_DS_2_VASc score was associated with a reduced risk of discontinuing dabigatran (HR: 0.78; 95% CI: 0.62–0.98).

### Healthcare resource use during follow-up

**Fig. 5:**
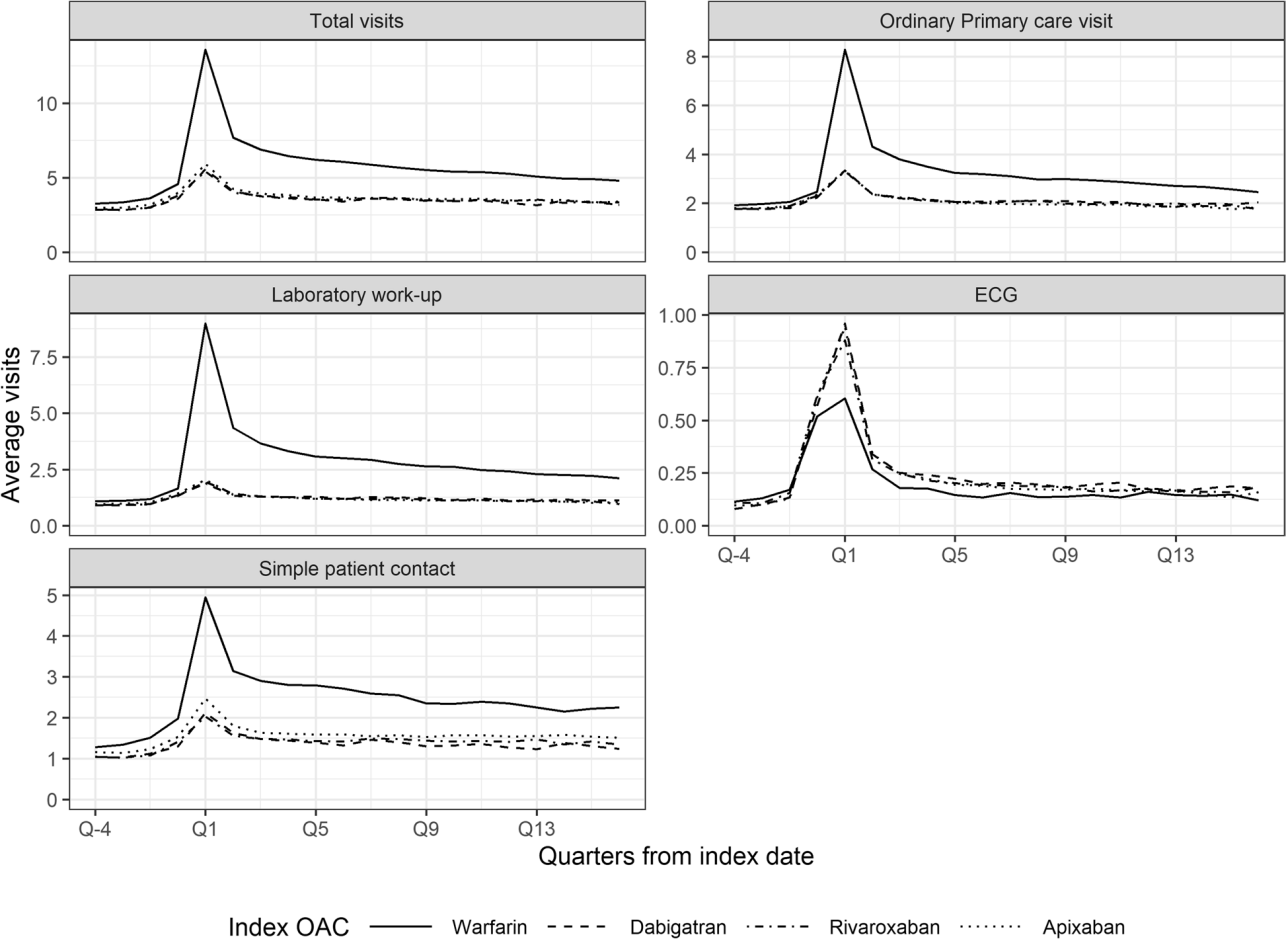
Healthcare Resource Use during Follow-up. Abbreviation: ECG = electrocardiogram; OAC = oral anticoagulant

The NOACs were similar in terms of number of general practitioner visits during follow-up, while warfarin differed from the NOACs (Fig. 5). The adjusted negative binomial regression found that NOACs had fewer total visits compared with warfarin: dabigatran (IRR: 0.66; 95% CI: 0.64–0.69), rivaroxaban (IRR: 0.66; 95% CI: 0.64–0.68), apixaban (IRR: 0.76; 95% CI: 0.74–0.78), and NOACs overall (IRR 0.73; 95% CI 0.71–0.75). There were also significantly fewer ordinary primary care visits: dabigatran (IRR: 0.70; 95% CI: 0.67–0.73), rivaroxaban (IRR: 0.69; 95% CI: 0.67–0.71), apixaban (IRR: 0.77; 95% CI: 0.75–0.80). There was also significantly less laboratory work up (dabigatran: IRR: 0.49; 95% CI: 0.47–0.51, rivaroxaban: IRR: 0.48; 95% CI: 0.46–0.49, and apixaban: IRR: 0.55; 95% CI: 0.54–0.57) and simple patient contact (dabigatran: IRR: 0.64; 95% CI: 0.60–0.67, rivaroxaban: IRR: 0.64; 95% CI: 0.61–0.66, and apixaban: IRR: 0.76; 95% CI: 0.73–0.79). The NOACs had more ECGs compared with warfarin: dabigatran (IRR: 1.18; 95% CI: 1.12–1.24) rivaroxaban (IRR: 1.16; 95% CI: 1.12–1.21), and apixaban (IRR: 1.34; 95% CI: 1.29–1.39).

## Discussion

In this longitudinal, population-based, nationwide Norwegian cohort study using primary care data, we found that patients with AF initiating OACs were older, had more comorbidities, and had a higher mean CHA_2_DS_2_-VASc score than untreated patients, although differences were small. Treatment with OACs for AF increased between 2010 and 2018, and the persistence of use was > 60% after four years. There was a shift from warfarin to NOACs during the study period, with 95% of new OAC-patients treated with NOACs in 2018. Using the standard dose rather than the reduced dose of the NOACs also became more prevalent during the study period, with 63%–81% being prescribed the standard dose in 2018. Switches from warfarin to NOACs, particularly apixaban, were common between 2015 and 2018, and discontinuation was most common among warfarin users and least common among apixaban users. The shift towards NOACs resulted in reduced primary care visits; NOAC users had a 27% reduced risk of primary care visits compared with warfarin users. Since previous studies focused on healthcare use in secondary care, this is an important addition to the current literature.

Our results, together with previous research [14, 19–21], show that warfarin use is decreasing in favour of NOACs. NOAC use appears to be extended to previously untreated parts of the population, since we have seen an increase in the proportion of patients with AF treated with an OAC over the years, driven by increased use of NOACs. However, our results show that there are still groups that are not treated according to guidelines, as the mean CHA_2_DS_2_-VASc score was more than 3 in the untreated female patients, contrary to guidelines that state those with a score ≥ 2 should receive treatment [2]. Focus on properly treating these patients should be a priority. We also observed that more patients were treated with standard-dose NOACs over the years. A possible reason for this is physicians becoming more aware of the importance of full (standard) dosing of NOACs. However, we cannot determine, based on registry data, whether patients were treated according to labels, since the registers lack data on variables such as kidney function and weight. Patients treated with apixaban experienced the fewest discontinuations and the least switching. This is in accordance with studies from other countries [28–31]. Treatment persistence is important to strive for, to avoid the patient burden of multiple switches and complications [32, 33].

We found that all NOACs had fewer total primary care visits compared with warfarin. More specifically, NOACs had fewer ordinary primary care visits, laboratory work up, and simple patient visits. The lack of regular monitoring requirements results in fewer primary care consultations for patients treated with NOACs, which results in saved resources in the primary care setting. This is in line with what was assumed when reimbursement discussions took place, although it is still important to follow-up with NOAC-treated patients [2]. However, even though the NOAC patients had fewer visits overall, we found they had more ECGs compared with warfarin. Previous studies have focused on healthcare use in secondary care and found indications of saved resources using NOACs over warfarin [22].

Strengths of this study include the use of real-world data from mandatory nationwide registries that minimised selection, participation, and recall bias. This also ensured a study population large enough for robust calculations. These advantages of nationwide registries are summarised in a recent position document from the European Heart Rhythm Association [34]. We were able to capture patients using OACs with a diagnosis of AF, and not VTE or any other condition, a challenge for similar studies based on registries where information on indication for treatment is unavailable. The use of primary care data enabled us to include the less severe cases managed in primary care only, increasing generalisability. Weaknesses include the fact that the study participants were largely white northern Europeans; this may limit the generalisability of the results outside these settings. Moreover, the registries do not supply information on relevant laboratory analyses, such as estimated glomerular filtration rate, cardiac troponins, erythrocyte count, thrombocyte count, or liver enzymes, or other important patient characteristics, such as body weight, lifestyle, or smoking habits [34].

## Conclusion

This study adds to the previous literature by assessing how the shift from warfarin to NOACs affected primary healthcare use. Our results showed that the shift from warfarin to NOACs in primary care in Norway has been efficient; of patients initiating an OAC in 2018, 95% were treated with NOACs. The persistence in OAC-treated patients was high, particularly for apixaban-treated patients. The shift also resulted in fewer general practitioner visits, which may lead to a reduced burden on patients as well as reduced societal costs. However, we found indications that some patients are still untreated.

## Supplementary Information

**Additional file 1.** Cohort Creation Flow Chart.

**Additional file 2.** Definitions of the Variables with Codes.

**Additional file 3.** Cumulative Incidence Curve for Switches from Apixaban to other Oral anticoagulants (graph with y-axis scale up to 10%).

## Data Availability

The data underlying this article were provided by the NorPD and KUHR registries by permission. Data will be shared on request to the corresponding author with permission from the International Ethics Committee and Registry holders.

## Abbreviations

AF: Atrial fibrillation
CHA_2_DS_2_-VASc: Cardiac failure or dysfunction, Hypertension, Age ≥ 75[Doubled], Diabetes, Stroke[Doubled]-Vascular disease, Age 65–74 and Sex category [Female]) score
CI: Confidence interval
ECG: Electrocardiogram
HAS-BLED: Hypertension, Age, Stroke, Bleeding tendency/predisposition, Labile international normalised ratios, Elderly age, Drugs or alcohol excess
HR: Hazard ratio
ICD: International Classification of Diseases
ICPC: International Classification of Primary Care
IRR: Incidence rate ratio
KUHR: Norwegian Primary Care Registry
NOAC: Non-vitamin K antagonist oral anticoagulants
NorPD: Norwegian Prescription Database
OAC: Oral anticoagulant
OR: Odds ratio
SMD: Standardised mean difference
VTE: Venous thromboembolism
