# Supplementary material for: Changes in primary care management of atrial fibrillation patients following the shift from warfarin to non-vitamin K antagonist oral anticoagulants: a Norwegian population based study
Source: BMC Prim Care. 2022 Aug 25;23:214. doi: 10.1186/s12875-022-01824-6 (PMC9404608; doi:10.1186/s12875-022-01824-6)
Supplement: Supplementary file 1 — Additional file 1. Cohort Creation Flow Chart. [file 12875_2022_1824_MOESM1_ESM.docx]

**Additional file 1. Cohort Creation Flow Chart**

**
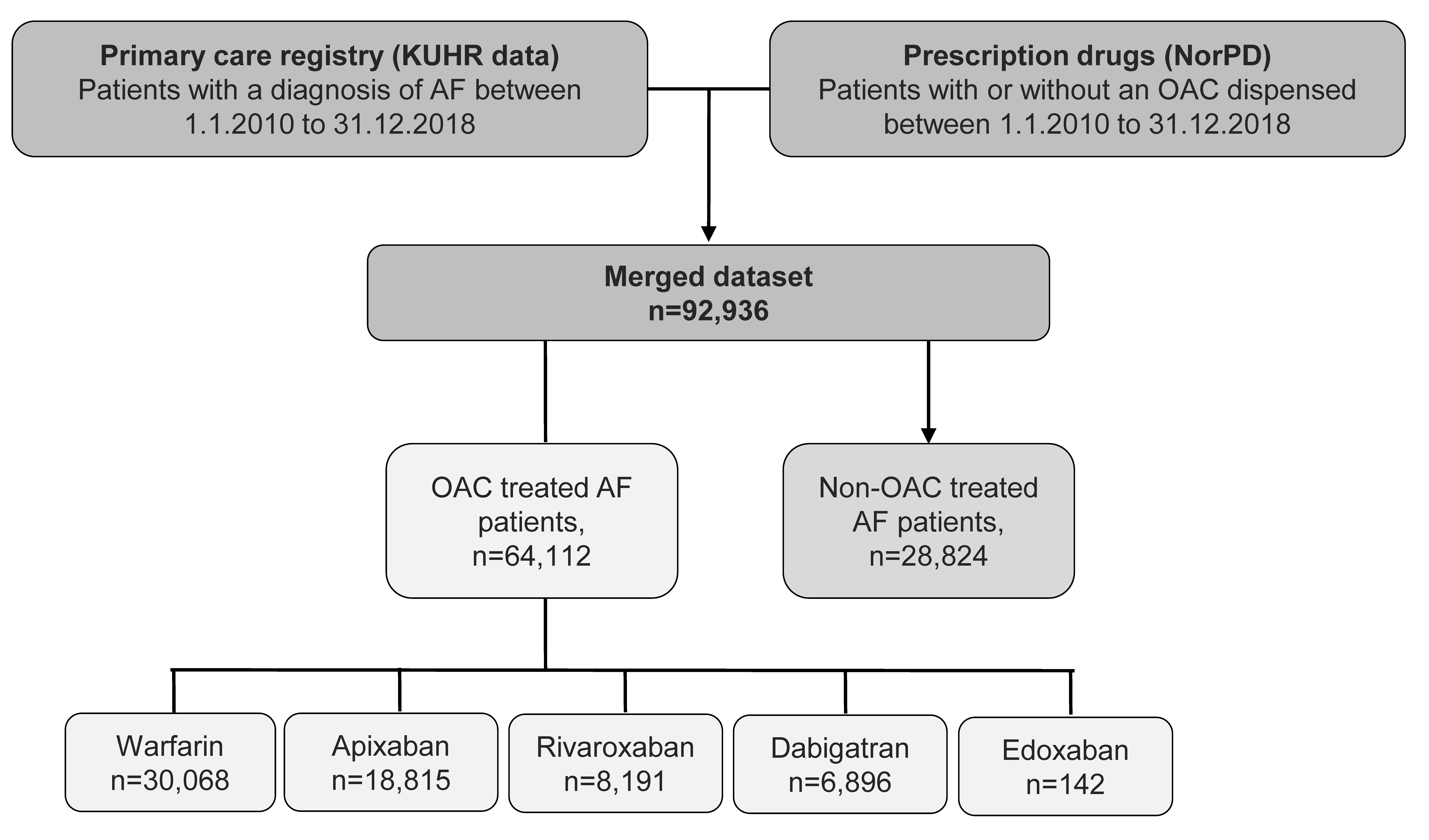
**

*Abbreviations*: AF= atrial fibrillation; KUHR = Norwegian Primary Care Registry; NorPD = Norwegian Prescription Database; OAC= oral anticoagulant
