# Supplementary material for: Changes in primary care management of atrial fibrillation patients following the shift from warfarin to non-vitamin K antagonist oral anticoagulants: a Norwegian population based study
Source: BMC Prim Care. 2022 Aug 25;23:214. doi: 10.1186/s12875-022-01824-6 (PMC9404608; doi:10.1186/s12875-022-01824-6)
Supplement: Supplementary file 2 — Additional file 2. Definitions of the Variables with Codes. [file 12875_2022_1824_MOESM2_ESM.docx]

**Additional File 2. Definitions of the Variables with Codes**

| **Variable** | **Data Source(s)** | **Operational Definition** | |
| --- | --- | --- | --- |
| Modified HAS-BLED score | KUHR and NorPD | - Hypertension.1 point - Renal impairment. 1 point - Liver impairment. 1 point - Prior stroke. 1 point - Prior major bleeding. 1 point - Age ≥ 65 years. 1 point - Therapy with NSAID (M01A) or anti-platelets (B01AC) in the previous year before index date. 1 point - Alcoholism. 1 point   **Total HAS-BLED score from 0 to 8** | |
| CHA_2_DS_2_VASc | KUHR and NorPD | - Chronic heart failure. 1 point - Hypertension. 1 point - Diabetes. 1 point - Age ≥75 years (2 points) or age 64–74 years (1 point) - Previous stroke, TIA or thromboembolism. 2 points - Vascular disease (e.g., peripheral artery disease, myocardial infarction, aortic plaque). 1 point - Sex (male=0 point, female=1 point)   **Total score 0 to 9** | |
| Co-morbidity index | KUHR and NorPD | - Age: ≤41 years (0 point), 41–50 years (1 point), 51–60 years (2 points), 61–70 (3 points), ≥71 years (4 points) - Myocardial infarction. 1 point - Congestive heart failure. 1 point - TIA or stroke. 1 points - Dementia. 1 point - COPD. 1 point - Connective tissue disease. 1 point - Prior major bleeding 1 point - Diabetes. 1 point - Moderate to severe chronic kidney disease. 2 points - Leukaemia. 3 points - Lymphoma. 3 points - Solid tumour. 3 points - Liver disease. 3 points   **Total score from 0 to 26** | |
| Valvular disease atrial fibrillation | KUHR | ICD-10/ICPC-2; I05/K71, I06/K71, I07/K71, I08/K83, I342/K83, Z952/K83 | |
| Hip/knee replacement surgery | KUHR | ICD-10: T84 or ICPC-2: A89 | |
| VTE | KUHR | ICD-10: I80 or ICPC-2: K94 | |
| Pregnancy | KUHR | ICD-10: Z33 or ICPC-2: W78 |  |
| Alcoholism | KUHR | ICD-10: E244, G312, G621, G721, I426, K860, O354, Z714, Z721, E52, K70, T51. ICPC: P15, P16 |  |
| Chronic kidney disease | KUHR | ICD-10: N18. ICPC: U99 |  |
| Congestive heart failure | KUHR | ICD-10: I50. ICPC: K77 |  |
| Hypertension | KUHR | ICD-10: I10-I15. ICPC-2: K85-K87 |  |
| Liver disease | KUHR | ICD-10: K70-K77. ICPC-2: D97 |  |
| Stroke | KUHR | ICD-10: I60-I64. ICPC-2: K90, K91 |  |
| TIA | KUHR | ICD-10: G459. ICPC-2: K89 |  |
| Myocardial infarction | KUHR | ICD-10: I21, I22. ICPC-2: K75 |  |
| Angina pectoris | KUHR | ICD-10: I20. ICPC-2: K74 |  |
| Peripheral artery disease | KUHR | ICD-10: I73.9. ICPC-2: K92 |  |
| Pulmonary embolism | KUHR | ICD-10: I26. ICPC-2: K93 |  |
| Prior major bleeding (critical organ) | KUHR | ICD-10: K920, K921, K922, K25x-K28x (with x as 0,2,4,6) K290, K625, I850, I983, I60x-I62x, N02, H431, K661, R042, R040, K250, N939. ICPC-2: D14, D15, D16, R06, A10 |  |
| Type 2 Diabetes | KUHR | ICD-10: E11. ICPC-2: T90 |  |
| Dementia | KUHR | ICD-10: F01-F03 and G30. ICPC-2: P70 |  |
| COPD | KUHR | ICD-10: J44. ICPC-2: R95 |  |
| Connective Tissue Disease | KUHR | ICD-10: M30-M36. ICPC-2: L99 |  |
| Leukaemia | KUHR | ICD-10: C91-C95. ICPC-2: B73 |  |
| Lymphoma | KUHR | ICD-10: C81-C85. ICPC-2: B72, B74 |  |
| Solid tumour | KUHR | ICD-10: C00-C97. ICPC-2: D74-D77, F74, H75, K72, L71, N74, R84-R85, S77, T71, U75-U77, X75-X77, Y77-Y78 |  |
| Warfarin | NorPD | ATC: B01A A03 |  |
| Dabigatran | NorPD | ATC: B01A E07 |  |
| Rivaroxaban | NorPD | ATC: B01A F01 |  |
| Apixaban | NorPD | ATC: B01A F02 |  |
| Edoxaban | NorPD | ATC: B01A F03 |  |
| NSAID | NorPD | ATC: M01A |  |
| Anti-platelet treatment, including low-dose aspirin | NorPD | ATC: B01AC |  |
| Per-oral antidiabetic drugs | NorPD | ATC: A10B |  |
| Acid secretory drugs | NorPD | ATC: A02B |  |
| Heparin | NorPD | ATC: B01AB |  |
| Anti-arrhythmic drugs class iii | NorPD | ATC: C01BD |  |
| Anti-hypertensives | NorPD | ATC: C02 |  |
| Diuretics | NorPD | ATC: C03 |  |
| Beta-blockers | NorPD | ATC: C07 |  |
| Calcium antagonists | NorPD | ATC: C08 |  |
| Renin-angiotensin system drugs | NorPD | ATC: C09 |  |
| Lipid-modifying drugs | NorPD | ATC: C10 |  |
| Insulin | NorPD | ATC: A10A |  |
| Ordinary primary care visit | KUHR | Claim codes: 2ad, 2ae, 2ak, 3ad, 11ad, 11ak |  |
| Laboratory work-up | KUHR | Claim codes: 701a, 710, 708b, 708d, 706f |  |
| ECG | KUHR | Claim code: 707 |  |
| Simple patient contact | KUHR | Claim codes: 1bd, 1be, 1bk, 1f, 1ad, 1ak |  |

Abbreviations: ATC = Anatomical Therapeutic Chemical; COPD = chronic obstructive pulmonary disease; ECG = electrocardiogram; ICD-10 = International Classification of Diseases, 10th Revision; ICPC-2 = International Classification of Primary Care, 2nd Edition; KUHR = Norwegian Primary Care Registry; NorPD = Norwegian Prescription Database; NSAID = non-steroidal anti-inflammatory drug; TIA = transient ischaemic attack; VTE = venous thromboembolism
