# Supplementary material for: Changes in primary care management of atrial fibrillation patients following the shift from warfarin to non-vitamin K antagonist oral anticoagulants: a Norwegian population based study
Source: BMC Prim Care. 2022 Aug 25;23:214. doi: 10.1186/s12875-022-01824-6 (PMC9404608; doi:10.1186/s12875-022-01824-6)
Supplement: Supplementary file 3 — Additional file 3. Cumulative Incidence Curve for Switches from Apixaban to other Oral anticoagulants (graph with y-axis scale up to 10%). [file 12875_2022_1824_MOESM3_ESM.docx]

**Additional file 3. Cumulative Incidence Curve for Switches from Apixaban to other Oral anticoagulants** (graph with y-axis scale up to 10%).
